# Toward Establishing Integrated, Comprehensive, and Sustainable Meningitis Surveillance in Africa to Better Inform Vaccination Strategies

**DOI:** 10.1093/infdis/jiab268

**Published:** 2021-09-01

**Authors:** Brenda Anna Kwambana-Adams, Adam L Cohen, Lee Hampton, Aquino Albino Nhantumbo, Robert S Heyderman, Martin Antonio, Andre Bita, Jason Mathiu Mwenda

**Affiliations:** 1 NIHR Global Health Research Unit on Mucosal Pathogens, Division of Infection and Immunity, University College London, London, United Kingdom; 2 World Health Organization Collaborating Centre for New Vaccines Surveillance, Medical Research Council Unit The Gambia at London School of Hygiene and Tropical Medicine, Fajara, Banjul, The Gambia; 3 Division of Global Health Protection, Center for Global Health, Centers for Disease Control and Prevention, Atlanta, Georgia, USA; 4 Gavi, The Vaccine Alliance, Global Health Campus, Geneva, Switzerland; 5 Laboratório Nacional de Referência de Microbiologia, Instituto Nacional de Saúde, Ministério da Saúde, Maputo, Mozambique; 6 Centre for Epidemic Preparedness and Response, London School of Hygiene and Tropical Medicine, London, United Kingdom; 7 Department of Infection Biology, Faculty of Infectious and Tropical Diseases, London School of Hygiene and Tropical Medicine, London, United Kingdom; 8 World Health Organization Regional Office for Africa, Brazzaville, Republic of Congo

**Keywords:** Surveillance, Acute bacterial meningitis, Sub-Saharan Africa, African meningitis belt, *Streptococcus pneumoniae*, *Neisseria Meningitidis*, vaccines

## Abstract

Large populations across sub-Saharan Africa remain at risk of devastating acute bacterial meningitis epidemics and endemic disease. Meningitis surveillance is a cornerstone of disease control, essential for describing temporal changes in disease epidemiology, the rapid detection of outbreaks, guiding vaccine introduction and monitoring vaccine impact. However, meningitis surveillance in most African countries is weak, undermined by parallel surveillance systems with little to no synergy and limited laboratory capacity. African countries need to implement comprehensive meningitis surveillance systems to adapt to the rapidly changing disease trends and vaccine landscapes. The World Health Organization and partners have developed a new investment case to restructure vaccine-preventable disease surveillance. With this new structure, countries will establish comprehensive and sustainable meningitis surveillance systems integrated with greater harmonization between population-based and sentinel surveillance systems. There will also be stronger linkage with existing surveillance systems for vaccine-preventable diseases, such as polio, measles, yellow fever, and rotavirus, as well as with other epidemic-prone diseases to leverage their infrastructure, transport systems, equipment, human resources and funding. The implementation of these concepts is currently being piloted in a few countries in sub-Saharan Africa with support from the World Health Organization and other partners. African countries need to take urgent action to improve synergies and coordination between different surveillance systems to set joint priorities that will inform action to control devastating acute bacterial meningitis effectively.

Meningitis is an inflammatory disease of the meninges, the membranous covering of the brain and spinal cord. Some of the most severe forms of the disease are caused by bacteria [1]. Acute bacterial meningitis (ABM) can kill within hours of disease onset and leave survivors with lifelong disabilities [2, 3]. The global estimates of meningitis stand at 2.8 million cases and approximately 300 000 deaths every year [4]. The African “meningitis belt” is a region spanning 26 contiguous countries, with an estimated 400 million people at risk of epidemic meningitis primarily caused by *Neisseria meningitidis* (the meningococcus) and *Streptococcus pneumoniae* (the pneumococcus) [5].

## ROLLOUT OF CONJUGATE VACCINES IN AFRICA

Conjugate vaccines that provide protection against ABM caused by *N. meningitidis* serogroup A (NmA), *Haemophilus influenzae* type b (Hib), and the pneumococcus have been rolled out across African countries [4, 6, 7]. To date, 39 of 47 countries in the World Health Organization (WHO) Regional Office for Africa (WHO/AFRO) have introduced the 10- or 13-valent formulations of the pneumococcal conjugate vaccine (PCV10 or PCV13) into their infant immunization programs [8]. The Hib conjugate vaccine has been introduced in all countries in WHO/AFRO as part of the pentavalent infant immunization vaccine over the last 20 years [6].

The WHO/AFRO Strategic Plan for Immunization 2014–2020 set 2 key objectives related to meningococcal meningitis control [9]: (1) rollout of meningococcal A protein–polysaccharide conjugate vaccine (MenAfriVac) campaigns in all 26 countries within the African meningitis belt and (2) introduction of MenAfriVac in the Expanded Programme on Immunisation (EPI) of at least 16 of the 26 countries in the African meningitis belt [10]. Between 2010 and 2018, MenAfriVac was rolled out through mass vaccination campaigns targeting individuals 1–29 years old across 24 of the 26 African meningitis belt countries [7]. To maintain high levels of herd immunity and sustain control of NmA, WHO recommends that countries that have conducted campaigns include MenAfriVac in their EPI. As of April 2021, a total of 12 of the 26 African meningitis belt countries had introduced MenAfriVac into their EPI, and 3 more countries (Togo, Benin, and Guinea Bissau) have planned introductions in 2021.

Group B *Streptococcus* (GBS) is an important cause of meningitis and sepsis in children <3 months of age [11]. As such, the development of GBS vaccines for maternal immunization has been prioritized by WHO, and a number of GBS vaccines are currently under development and at various stages of clinical trials [12].

## THE IMPORTANCE OF MENINGITIS SURVEILLANCE

Meningitis surveillance provides health-related data to inform vaccination programs and is useful for monitoring the impact of public health practice. Effective meningitis surveillance entails systematic and continuous collection and interrogation of data to; (1) identify priority pathogens and populations for vaccination, (2) guide introduction of new vaccines, (3) detect outbreaks, (4) describe changes in disease epidemiology over time, including the impact of vaccination, (5) monitor antimicrobial resistance, and (6) describe sequelae and long-term disability among meningitis survivors [9].

Disease surveillance is one of the 5 pillars in the WHO road map to defeating meningitis by 2030 [13]. The importance of surveillance is emphasized by both the Addis Declaration on Immunisation (2012) endorsed by heads of state at the 28th African Union Summit, and the Immunisation Agenda 2030, endorsed by the World Health Assembly in 2020 [14]. The need for comprehensive and robust meningitis surveillance systems is great in Africa. The continent is currently faced with the emergence of meningococcal serogroup C, W, and X strains with epidemic potential [15–18], which threaten the success of meningococcal meningitis control achieved through the MenAfriVac programs [15–18]. 

Currently available polyvalent meningococcal vaccines (comprising serogroups A, C, Y, and W) are not affordable, and their use in Africa has been restricted to reactive vaccination campaigns [19]. A pentavalent meningococcal conjugate vaccine targeting serogroups A, C, Y, W, and X is in the pipeline; however, data from surveillance systems are needed to guide vaccine strategy and implementation once the vaccine becomes available [20]. Robust surveillance data are also required to monitor the success of the immunization programs and establish whether herd immunity protects unvaccinated populations. There also remains a significant burden of epidemic meningitis caused by serotype 1 pneumococcus, which is covered by both PCV10 and PCV13. Therefore, there are questions around optimal PCV scheduling and the long-term benefits of a booster dose for enhancing herd protection and accelerating control of serotype 1 and other PCV serotypes, particularly in outbreak-prone regions [21]. For GBS, accurate data on the disease burden and circulating serotypes are needed to inform vaccine development and support the rollout of effective vaccines once they become available [12]. Addressing these critical questions around optimal vaccination strategy and scheduling is largely dependent upon data generated through effective surveillance.

## CURRENT MENINGITIS SURVEILLANCE SYSTEMS AND THEIR LIMITATIONS

Although sub-Saharan Africa has the largest burden of meningitis globally, most countries have meningitis surveillance systems that are weak, poorly integrated, and rely heavily on external financing and technical assistance [9]. Of the 47 WHO/AFRO member states, 39 allocate less than 10% of their routine immunization expenditure to surveillance activities, with an even smaller proportion spent on meningitis surveillance specifically [9]. This challenge is compounded by countries running several meningitis surveillance systems in parallel, further stretching the already limited resources available [9, 22]. Surveillance efforts are further undermined by often intermittent or incomplete laboratory identification of pathogens. Although there are a few exceptions [23], there is limited intracountry and intercountry synergy in meningitis surveillance programs, despite the potential benefits of regional cooperation in tackling meningitis epidemics and endemic disease.

The Integrated Disease Surveillance and Response (IDSR) system was established in 1998 by WHO/AFRO in partnership with technical partners. This system uses standardized tools to collect aggregated data for multiple diseases and was adopted by all AFRO member states [24]. The Enhanced Meningitis Surveillance system was built on the IDSR framework and implemented in 24 African meningitis belt countries in 2004 to enable early detection, confirmation, and response to meningitis outbreaks [25]; the system collects clinical, demographic, and laboratory data from suspected meningitis cases, which are reported weekly.

In 2001, the WHO-supported African Paediatric Bacterial Meningitis (PBM) surveillance network was established [22]. The PBM network supports hospital-based sentinel surveillance of meningitis among children less than 5 years old across 33 countries in WHO/AFRO. In 2008, the PBM Network became part of the global invasive bacterial vaccine-preventable disease (IB-VPD) network. The IB-VPD surveillance platform collects data on the epidemiology of invasive bacterial vaccine-preventable diseases (VPDs) and evaluates vaccine impact [26]. The PBM and IB-VPD networks have made significant efforts to standardize data collection as well as clinical and laboratory protocols in participating states.

Leveraging the Enhanced Meningitis Surveillance system, the MenAfriNet consortium successfully established case-based meningitis surveillance in 5 meningitis belt countries (Burkina Faso, Chad, Mali, Niger, and Togo) in 2014 [27]. Case-based surveillance in these countries provided data on the epidemiologic trends of meningitis following the implementation of MenAfriVac and PCV. In the next phase of MenAfriNet, the focus is on further strengthening case-based surveillance in Burkina Faso and Niger to support introduction of the pentavalent meningococcal vaccine, with regional support for other countries in the African meningitis belt, including Chad, Mali, and Togo [23].

Other types of meningitis surveillance operational in the WHO/AFRO region are the notifiable disease surveillance system and other laboratory-based surveillance systems. South Africa, for instance, which did not implement the IDSR system, uses the notifiable disease surveillance system and the Group for Enteric, Respiratory and Meningeal Disease Surveillance in South Africa (GERMS-SA) to track bacterial meningitis [28, 29].

Although data collected through all these surveillance programs were used to support the implementation of vaccines and monitor vaccine impact, there remain large gaps in the understanding of meningitis across most African countries, particularly beyond early childhood and the 3 main bacterial pathogens (pneumococcus, meningococcus and *H. influenzae*) [30].

## THE CASE FOR AN INTEGRATED MENINGITIS SURVEILLANCE SYSTEM

The rapidly changing epidemiology of VPDs and the evolving vaccine landscape coupled with multiple meningococcal outbreaks caused by non-A serogroups call for urgent action to strengthen meningitis surveillance in sub-Saharan Africa. The PBM and Global IB-VPD surveillance networks leveraged funding and resources through Gavi, the Vaccine Alliance, and the Global Polio Eradication Initiative; however, the funding available is declining [9]. The lack of harmonization in the current meningitis surveillance programs within and across countries means that there are significant redundancies and cost inefficiencies.

Integration of existing meningitis surveillance systems will be key to achieving sustainable, comprehensive systems that are synergistic, efficient, and cost-effective. As such, WHO recommends the integration of PBM, enhanced, and case-based meningitis surveillance programs that currently run in parallel in most countries in WHO/AFRO. Furthermore, meningitis surveillance should not be in a separate silo; instead, it should be integrated into the VPD surveillance systems supported by strong laboratories with the capacity to detect cases and outbreaks, and generate useable information to guide outbreak preparedness and responses, immunization, program optimization, and vaccine policy as outlined in recent WHO guidance documents [9, 31].

The 6 key components for efficient, comprehensive and integrated surveillance of VPDs, as proposed in the WHO/AFRO investment case are as follows: 

1. Governance and management: The establishment of country-owned and country-funded surveillance systems integrated within national VPD surveillance. This will entail greater commitment, ownership and leadership with accountability from the member states at the supranational, national, subnational, and district levels.

2. Standards for surveillance (harmonization): Setting up standardized guidelines and practices for meningitis surveillance and other VPDs. High-quality management will be needed to monitor and evaluate compliance with guidelines, standard case definitions, procedures, and data collection tools.

3. Laboratory capacity and networks: Leveraging well-established laboratory networks for other VPDs, such as measles, yellow fever, rotavirus, and polio as much as possible. These networks seek to ensure that laboratories are adequately equipped, have staff trained according to standardized protocols, maintain quality controls and have accreditations. This is particularly important for meningitis caused by the pneumococcus, meningococcus, *H. influenzae* and GBS, for which polymerase chain reaction (PCR)–based serotyping/serogrouping capacity is essential.

4. Specimen management: Maintaining high quality standards for the collection, storage and transportation of cerebrospinal fluid (CSF) specimens, according to standard operating procedures. This requires the use of the appropriate specimen collection tools (eg, lumbar puncture kits), storage media (eg, Trans-Isolate (TI) medium), and preservation of specimens for molecular analysis. Establishing efficient supply chains for specimen management is critical, especially during meningitis epidemics.

5. Detection and response: Drawing up and updating national epidemic preparedness plans for the rapid detection and confirmation of meningitis outbreaks. Preparedness plans should include risk-mapping, assessment and mitigation plans, staff mobilization, community awareness, communication strategies, and evaluation of emergency response performance. These plans should be adaptive to unexpected changes and reviewed regularly. 

6. Surveillance process, data management and analysis, and reporting: Effective and timely responses depend on high-quality information (accurate and complete) to be collected at all stages, including case detection, notification, investigation, and confirmation. Health facilities are the primary case-reporting sites; as such, training and sensitization programs need to be inclusive, involving health workers, disease surveillance officers, and the communities they serve.

## THE CASE FOR EFFECTIVE USE OF SURVEILLANCE DATA TO INFORM EPI DECISIONS

Nearly all countries in WHO/AFRO that have introduced PCV, MenAfriVac and Hib conjugate vaccinee have done so with financial support from Gavi, The Vaccine Alliance. In preparation for the pentavalent meningococcal vaccine, which is likely to become available in the next 2–4 years, the Gavi board approved the expansion of the existing meningococcal program. However, this approval will be contingent on an evidence-based technical recommendation on vaccination strategy to identify the target age groups, duration of protection, and priority communities. Hence, compared with the current Gavi vaccine-support model for MenAfriVac, Gavi-supported implementation of the multivalent meningococcal vaccine in routine immunization and campaigns will be more narrowly targeted in terms of both geographic areas and age groups covered. It is expected that the recommended vaccination strategy will vary among countries in the African meningitis belt.

Member states will need to consider these approval requirements as they prepare for the rollout of the pentavalent meningococcal vaccine. Countries will need to generate disease burden data through surveillance, including serogroups and genotypes of the causative pathogens. These data will be necessary to identify high-priority populations and age groups as well as guide vaccination strategy (mass vaccination campaigns, routine vaccination, or reactive vaccination).

## PILOTING MENINGITIS SURVEILLANCE INTEGRATION

At the 17th Annual Meeting on Surveillance, Preparedness and Response to Meningitis Outbreaks in Africa and the seventh meeting of MenAfriNet Partners, held virtually in December 2020, the feasibility of integrating meningitis surveillance and adapting recent technologies to improve surveillance in Africa was discussed. Several countries expressed interest in exploring the possibility of expanding meningitis surveillance to collect data on all ages using a “one meningitis approach”. These concepts are being piloted in Ghana, Togo, Nigeria, Mozambique, Burundi, and Tanzania with support from WHO and partners. The US Centers for Disease Control and Prevention and Takeda Pharmaceutical Company are also funding pilots of comprehensive surveillance approaches in 14 countries in the WHO/AFRO Region.

During this meeting, Mozambique shared experiences and outlined ongoing efforts to expand and integrate meningitis surveillance. With the support of Gavi, Mozambique introduced PCV10 into its EPI in April 2013 [32]. PCV10 was replaced by PCV13 nationwide in May 2019, with a phased rollout that started in December 2017 in the north of the country. Instituto Nacional de Saúde (INS) has been conducting surveillance for meningitis since 2013 at 3 sentinel sites. In 2018 a surveillance evaluation conducted by the Centers for Disease Control and Prevention and WHO/AFRO in collaboration with INS revealed several gaps in case identification, specimen collection, storage, and transport. Therefore, in November 2019, Mozambique INS embarked on the phased expansion of its meningitis surveillance system to include adults as part of integrated surveillance.

## NEW TECHNOLOGIES AND INNOVATIONS

Improving meningitis diagnosis is one of the key priorities in the WHO road map for defeating meningitis by 2030 [14]. Implementation of new diagnostic innovations can greatly improve meningitis surveillance in WHO/AFRO, where most laboratories have limited microbiologic capacity.

Molecular tools offer additional diagnostic power alongside traditional culture methods in that they are rapid, highly sensitive, and specific. Molecular tools can detect pathogens in archived specimens and patients who have received prior antibiotic treatment, are high throughput, and can be used to type pathogens. Although quantitative PCR (qPCR) is used to detect pathogens in the Africa PBM surveillance [22], MenAfriNet surveillance activities [27] and meningococcal carriage surveys in the African meningitis belt [33], these tools are not yet widely used for meningitis diagnosis across WHO/AFRO. QPCR is performed routinely on CSF and/or blood collected from suspected cases of meningitis in many high-income countries, including the United Kingdom, and has been shown to increase case confirmation [34]. The adoption of molecular tools for ABM detection would circumvent some of the challenges posed by limited microbiologic capacity for CSF and blood cultures. Improving access to qPCR-based meningitis diagnostic tools should be an important part of efforts to improve VPD surveillance in WHO/AFRO Region.

The TaqMan Array Card (TAC) is a qPCR-based detection platform that is useful for the simultaneous and accurate detection of multiple bacterial, viral, protozoan, and fungal pathogens, using very small amounts of clinical specimens. Kwambana-Adams et al [30] demonstrated the utility of a TAC platform that significantly enhanced the diagnosis of meningitis pathogens compared to conventional molecular tools among West African children <5 years of age with suspected meningitis, who were identified through the PBM network.

Rapid diagnostic tests through immunochromatography and latex agglutination testing are simple to use, can provide results in <2 hours, and have the potential to enhance meningitis surveillance in WHO/AFRO. Although meningitis rapid diagnostic tests have been developed, most, including currently available latex agglutination tests, have been shown to have limited utility and reliability for meningitis diagnosis in low-resource settings [35]. However, new rapid tests are showing promise and may become widely available in the coming years. If that happens, they could play a very important role in improving meningococcal surveillance in the WHO/AFRO.

Pathogen whole-genome sequencing (WGS) is becoming increasingly accessible and can be used in combination with epidemiologic data to type strains, infer the spread of antimicrobial resistance genes, and identify transmission networks, particularly in outbreak scenarios. WGS was used to demonstrate that the NmC strain that caused the most recent large meningitis outbreaks in Niger [16] and Nigeria [15] belonged to the sequence type 10217. This outbreak NmC strain had emerged from a benign commensal organism that acquired capsule and virulence genes that confer epidemic potential [18].

WGS has relied on the isolation of viable bacteria, so that large amounts of DNA can be purified. However, the major challenge to this approach is that bacterial culture and storage capacity tends to be limited, especially in outbreak settings in Africa. Metagenomic approaches have been used to characterize meningitis during outbreaks [36–38] and during surveillance [39]. In the future and with further validation, meningitis surveillance could include the use of portable real-time sequencing platforms such as the Oxford Nanopore Technologies MinION which can be used to sequence nucleic acids from CSF [40] at the bedside, even in remote areas. These innovative technologies in surveillance programs will allow real-time detection, serotyping/serogrouping, genotyping, and antimicrobial resistance prediction of meningitis pathogens, which could be a game changer for outbreak responses in Africa.

Innovations in web-based data collection and sharing tools mean that data can be shared more quickly and securely. The use of platforms that allow interrogation and management of data collection in real time can enhance overall data quality (completeness and accuracy), which is at the core of any successful surveillance system. One such innovation which is being explored for meningitis sentinel surveillance is the Research Electronic Data Capture (REDCap) data collection instrument offered by the Johns Hopkins Biostatistics Center [41]. The REDCap system is more secure than most, can be accessed from any device with an internet connection, and maintains audit trails of all activity, and data collected can be stored independently by each stakeholder. Other systems, including District Health Information Software 2 and the Surveillance Outbreak Response Management and Analysis System have similar features. These data collection instruments can enhance the performance of meningitis surveillance programs, which often involve several stakeholders at various levels with data sharing in multiple directions.

## CARRIAGE SURVEYS AS COMPLEMENTARY TO ROUTINE SURVEILLANCE

Upper airway carriage of the pneumococcus and meningococcus is a prerequisite for disease and onward transmission across individuals [42]. Carriage surveys can be useful tools to measure vaccine effectiveness in clinical trials, monitor vaccine impact on circulating strains after implementation, and provide information on pathogen circulation, especially where the disease burden is difficult to measure [43].

The African Meningococcal Carriage Consortium (MenAfriCar) conducted a series of sequential meningococcal carriage surveys in urban and rural populations across 7 countries in the African meningitis belt, before and after the rollout of MenAfriVac vaccination campaigns [44, 45]. Although meningococcal carriage prevalence was 3.4%, relatively lower than in high-income countries [46], this study showed complex and highly dynamic carriage patterns. The MenAfriCar study showed a 98% decline in the carriage prevalence of NmA, comparable to the 94% reduction in NmA disease in vaccinated communities [45]. Carriage surveys were also used to guide Hib implementation in The Gambia and have been used to monitor vaccine impact on circulating strains after implementation [47, 48].

In addition to monitoring vaccine impact, carriage surveys can also be used to identify risk factors for exposure and reservoirs of outbreak strains [49, 50]. For instance, a carriage survey conducted during a meningococcal serogroup C (NmC) outbreak among university campus residents in the United Kingdom [51], revealed that the campus bars were linked to the carriage and transmission of the outbreak NmC strain [51]. Carriage surveys are therefore also useful to inform prevention and control strategies during ABM outbreaks.

Carriage surveys can also be useful for the isolation, antimicrobial susceptibility testing, and genomic sequencing of circulating strains in situations where microbiologic culture from invasive sites (CSF and blood) is difficult or not possible. As WHO/AFRO member states move toward integrated meningitis surveillance, carriage surveys could be employed to complement disease surveillance activities.

## Conclusions

Effective meningitis surveillance is the cornerstone of disease control and elimination. As such, it is one of the 5 overlapping pillars to defeat meningitis, as outlined in the WHO road map [13]. With increasing pressures from emerging infections and the implementation of new vaccines, the need for WHO/AFRO countries to establish effective meningitis surveillance systems has never been greater. Here, we have made the case that WHO/AFRO member states need to integrate the current meningitis surveillance systems to attain and maintain high-quality and broad-based surveillance for meningitis. The establishment of country-owned, comprehensive and sustainable meningitis surveillance systems hinges on effective integration and harmonization of meningitis surveillance within and across regions. Countries need to put plans into action to improve synergies and coordination between surveillance and immunization programs and to set joint priorities informing action to effectively control meningitis.

## Notes

***Acknowledgment.*** We acknowledge Cassandra Quintanilla from Gavi, The Vaccine Alliance, for her contributions to the concept of integrated meningitis surveillance and providing technical guidance.

***Disclaimer.*** The findings and conclusions in this report are those of the authors and do not represent the official position of the World Health Organization, the US Centers for Disease Control and Prevention, or Gavi, The Vaccine Alliance. The views expressed in this publication are those of the author(s) and not necessarily those of the National Institute for Health Research or the Department of Health and Social Care.

***Supplement sponsorship.*** This supplement is sponsored by the World Health Organization and the U. S. Centers for Disease Control and Prevention.

***Financial support.*** Funding for invasive bacterial vaccine-preventable disease surveillance was provided by Gavi, The Vaccine Alliance, through support to the World Health Organization.

***Potential conflicts of interest.*** All authors: No reported conflicts. All authors have submitted the ICMJE Form for Disclosure of Potential Conflicts of Interest. Conflicts that the editors consider relevant to the content of the manuscript have been disclosed.
